# Point of tangency between coronavirus disease and endothelial injury

**DOI:** 10.1186/s12245-021-00403-3

**Published:** 2021-12-20

**Authors:** So Sampei, Hideshi Okada, Hiroyuki Tomita, Akio Suzuki, Takahide Nawa, Shinji Ogura

**Affiliations:** 1 Department of Emergency and Disaster Medicine, Gifu University Graduate School of Medicine, 1-1 Yanagido, Gifu, 501-1194 Japan; 2 Department of Internal Medicine, Seiryu Hospital, Gifu, Japan; 3 Department of Tumor Pathology, Gifu University Graduate School of Medicine, Gifu, Japan; 4 Department of Pharmacy, Gifu University Hospital, Gifu, Japan

**Keywords:** COVID-19, SARS-CoV-2, RT-PCR, Glycocalyx

To the Editor,

We report on a 74-year-old patient with severe pneumonia who had negative results on the first two reverse transcription-polymerase chain reaction (RT-PCR) examinations (at hospital days 1 and 4). His medical history and computed tomography findings strongly suggested coronavirus disease-2019 (COVID-19) (Fig. 1). Moreover, he had been undergoing hemodialysis for 5 years for chronic renal failure due to diabetic nephropathy. The patient tested positive on the third RT-PCR examination and was diagnosed as having a COVID-19 infection on hospital day 7.

**Fig. 1:**
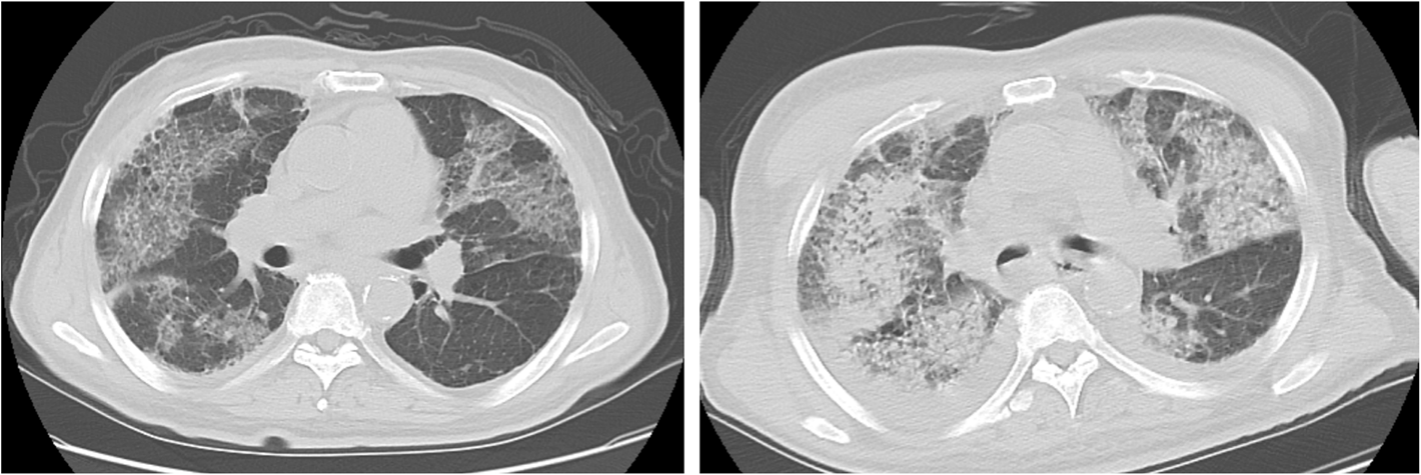
Lung computed tomography at hospital days 1 (Left Panel) and 4 (right panel). Although these findings strongly suggested COVID-19, both of RT-PCR examinations resulted negative

RT-PCR, which targets selected genes of the severe acute respiratory syndrome-coronavirus-2 (SARS-CoV-2) RNA, has been the primary diagnostic tool for COVID-19. However, several reports have criticized this method, particularly its sensitivity [1], as false-positive or false-negative results are commonly observed and are caused by factors such as sampling errors, RT-PCR inaccuracy, and low amount of viral particles. In addition, it was recently reported that for patients with type 2 diabetes PCR testing needs to be repeated to diagnose COVID-19 [1].

It is thought that the primary target of SARS-CoV-2 is lung epithelial cells. Conversely, previous post-mortem analysis showed viral elements within endothelial cells and accumulation of inflammatory cells, with evidence of endothelial and inflammatory cell death [2]. It is probable that endotheliitis occurs subsequent to systemic inflammation and thrombosis caused by initial lung epithelial cell infection in COVID-19, despite non-direct viral infection of the endothelial cells; however, the underlying mechanisms remain incompletely understood.

SARS-CoV-2 infection has been associated with endothelial glycocalyx injury [3]. The endothelial glycocalyx coats the healthy vascular endothelium and is essential in microvascular and endothelial physiology as it regulates leukocyte adhesion and migration while inhibiting intravascular thrombosis. In a healthy person, the receptors related to adhesion on the endothelium surface are covered by glycocalyx. However, when the endothelial glycocalyx is reduced, these receptors are exposed on the surface of the endothelium. The reduced thickness of the endothelial glycocalyx is more pronounced in patients with diabetes mellitus, cardiovascular disease, hyperlipidemia, and hypertension as well as in the elderly and in smokers. These are also the underlying diseases that can increase the severity of COVID-19 [3]. Furthermore, the endothelial glycocalyx is reportedly injured in patients with COVID-19 [4].

A previous study suggested that the endothelial glycocalyx was already injured in diabetic patients and that widespread inflammatory cell migration aggravated inflammation [5]. Thus, the inflammation can become severe even with a low viral load. A similar phenomenon may occur in patients with an endothelial glycocalyx injured by an underlying disease not limited to diabetes. However, this is circumstantial and indirect evidence.

The direct relationship between COVID-19 and endothelial glycocalyx injury requires further investigation. Future studies are needed to establish the diagnostic and therapeutic importance of the endothelial glycocalyx.

## Data Availability

The datasets used and/or analyzed during the current study are available from the corresponding author on reasonable request.

## Abbreviations

COVID-19: Coronavirus disease-2019
RT-PCR: Reverse transcription-polymerase chain reaction
SARS-CoV-2: Severe acute respiratory syndrome-coronavirus-2

## References

[1] Salerno S, Zhao Z, Prabhu Sanakar S, Salvatore M, Gu T, Fritsche LG, Lee S (2021). Patterns of repeated diagnostic testing for COVID-19 in relation to patient characteristics and outcomes. J Intern Med.

[2] Varga Z, Flammer AJ, Steiger P, Haberecker M, Andermatt R, Zinkernagel AS, Mehra MR, Schuepbach RA, Ruschitzka F, Moch H (2020). Endothelial cell infection and endotheliitis in COVID-19. Lancet.

[3] Okada H, Yoshida S, Hara A, Ogura S, Tomita H (2021). Vascular endothelial injury exacerbates coronavirus disease 2019: the role of endothelial glycocalyx protection. Microcirculation.

[4] Suzuki K, Okada H, Tomita H, Sumi K, Kakino Y, Yasuda R, Kitagawa Y, Fukuta T, Miyake T, Yoshida S, Suzuki A, Ogura S (2021). Possible involvement of Syndecan-1 in the state of COVID-19 related to endothelial injury. Thromb J.

[5] Sampei S, Okada H, Tomita H, Takada C, Suzuki K, Kinoshita T, Kobayashi R, Fukuda H, Kawasaki Y, Nishio A, Yano H, Muraki I, Fukuda Y, Suzuki K, Miyazaki N, Watanabe T, Doi T, Yoshida T, Suzuki A, Yoshida S, Kushimoto S, Ogura S (2021). Endothelial glycocalyx disorders may be associated with extended inflammation during endotoxemia in a diabetic mouse model. Front Cell Dev Biol.

